# The clinical response to vemurafenib in a patient with a rare *BRAF*^*V600DK601del*^ mutation-positive melanoma

**DOI:** 10.1186/1471-2407-14-727

**Published:** 2014-09-29

**Authors:** Stéphanie Trudel, Norbert Odolczyk, Julie Dremaux, Jérôme Toffin, Aline Regnier, Henri Sevestre, Piotr Zielenkiewicz, Jean-Philippe Arnault, Brigitte Gubler

**Affiliations:** Laboratoire d’Oncobiologie moléculaire, Centre Hospitalier Universitaire Amiens Picardie, F-80054 Amiens, France; EA 4666 Lymphocyte Normal et Pathologique et Cancers, Université de Picardie Jules Verne, F-80054 Amiens, France; Department of Bioinformatics, Institute of Biochemistry and Biophysics, Polish Academy of Sciences, 02-106 Warsaw, Poland; Laboratoire d’Anatomie et Cytologie Pathologiques, Centre Hospitalier Universitaire Amiens Picardie, F-80054 Amiens, France; Laboratory of Plant Molecular Biology, Faculty of Biology, Warsaw University, 02-106 Warsaw, Poland; Service de Dermatologie, Centre Hospitalier Universitaire Amiens Picardie, F-80054 Amiens, France

**Keywords:** Metastatic melanoma, Rare *BRAF* mutations, Monoallelic mutation, V600E, V600DK601del, Crystal structures, BRAF inhibitor, Vemurafenib

## Abstract

**Background:**

Mutations in the activation segment of the v-raf murine sarcoma viral oncogene homolog B (*BRAF)* gene are present in approximately 50% of melanomas. The selective BRAF inhibitor vemurafenib has demonstrated significant clinical benefits in patients with melanomas harboring the most common mutations (V600E, V600K and V600R). However, the clinical activity of BRAF inhibitors in patients with rare mutations of codon 600 and the surrounding codons has not been documented.

**Case presentation:**

We used the BRAF inhibitor vemurafenib to treat a patient presenting a rare p.V600_K601delinsD-mutated melanoma. An objective response was evidenced by two months of progression-free survival. By cloning and sequencing *BRAF* exon 15, we confirmed that a dual mutation was present on a single allele and thus resulted in a *BRAF*^*V600DK601del*^ mutant protein. We also performed an *in silico* crystal structure analysis of the mutated protein, in order to characterize the nature of the putative interaction between vemurafenib and the mutant protein.

**Conclusion:**

This clinical experience suggests that (i) patients with *BRAF*^*V600DK601del*^-mutation-positive melanoma can be treated successfully with the oral BRAF inhibitor vemurafenib and (ii) molecular screening in this context should encompass rare and complex mutations.

## Background

Melanoma is the malignancy with the highest prevalence of *BRAF* gene mutations. The most frequent *BRAF* mutation is a substitution at the second position of codon 600 (G**T**G > G**A**G), c.1799 T > A), which results in an amino acid change from valine (V) to glutamic acid (E) (p.V600E). In the initial study by Davies et al. [1], the p.V600E mutation accounted for more than 90% of *BRAF* mutations. Although the high frequency of *BRAF* mutations (and particularly the p.V600E mutation) in melanoma has been confirmed in all subsequent studies (for a review, see [2, 3]), its incidence is usually somewhat lower than initially reported. In fact, large, recent studies have shown that the p.V600E genotype is not as prevalent as expected and that p.V600K and p.V600R respectively account for 17%-22% and 3%-4% of the *BRAF* mutation-positive melanoma population [4, 5]. Moreover, less frequent mutations were subsequently reported by other researchers, including other codon 600 mutations (p.V600E2 and p.V600D/M/G) and mutations in codons in the vicinity of codon 600 (such as p.D594N/V, p.G596R, p.L597R/V/S/Q and p.K601E/N) [4–11]. These “orphan” non-p.V600E/K/R mutations account for approximately 6% of all mutations reported for melanoma in the COSMIC database [12].

In terms of structural and functional aspects, the activation segment of BRAF’s kinase domain binds to the P-loop via predominantly hydrophobic interactions. In particular, the valine at position 600 (within the activation segment) is thought to hold the protein in an inactive conformation [13]. In the *BRAF*^*V600E*^ mutant protein, the hydrophobic valine is replaced by a glutamic acid residue, which disrupts the interaction between the activation segment and the P-loop and leads to a marked increase in kinase activity [13].

Advances in the molecular characterization of melanoma and *BRAF*^*V600E*^ mutant protein and better knowledge of the BRAF signaling cascade have enabled the development of therapeutic BRAF-kinase inhibitors that target the constitutively active mutant BRAF protein. Vemurafenib (formerly PLX-4032) was specifically designed to target BRAF protein kinase with the oncogenic mutation p.V600E [14] and received marketing approval in the USA and Europe in 2011. The drug is licensed as a monotherapy for patients with advanced melanoma harboring a *BRAF*^*V600*^ mutation.

In various prospective studies, vemurafenib has demonstrated significant clinical benefits in patient with p.V600E BRAF-mutation-positive metastatic melanoma. The response rate (according to the Response Evaluation Criteria In Solid Tumors (RECIST) criteria) is approximately 50%, and vemurafenib treatment is associated with prolonged progression-free survival (PFS) and overall survival when compared with the reference standard treatment (dacarbazine-based chemotherapy) [15–17]. A few reports have suggested that melanoma harboring a p.V600K or a p.V600R mutation may also respond to vemurafenib therapy [17–19]. However, it is not yet clear whether BRAF inhibitors are effective in patients with rare-mutation-positive melanoma. With the exception of scarce *in vitro* data, there is no evidence to suggest that selective BRAF inhibitors show clinical activity against orphan non-p.V600E/K/R mutations and so clinical efficacy is not well established.

Here, we report the case of a patient with metastatic melanoma harboring a rare and complex *BRAF* mutation. A course of vemurafenib therapy was associated with two months of PFS. We also performed molecular characterization of the mutation and an *in silico* crystal structure analysis of the mutated BRAF domain.

## Case presentation

### Clinical course and pathological features

**Figure 1:**
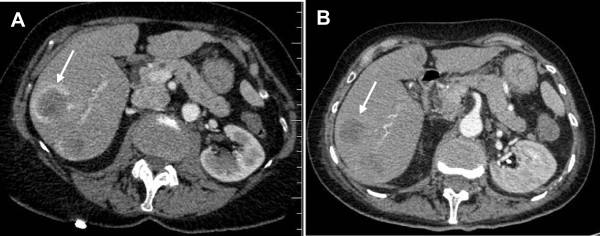
**Computed tomography.** The figures shows the initial CT scan of the liver (performed at the time that the metastatic stage of the disease was diagnosed) **(A)** and a CT scan acquired 4 weeks after the initiation of vemurafenib treatment **(B)**. The metastasis is indicated by an arrow. Comparison of the two scans reveals a partial regression of the metastatic tumor.

In February 2007, a 66 year-old woman was diagnosed as having a 2.35 mm cutaneous nodular thoracic melanoma and underwent complete excision with 2 cm margins. In March 2012, routine monitoring with computed tomography (CT) revealed two metastatic pulmonary nodes in the left upper and lower lobes. The subsequent pathological diagnosis (based on analysis of the two wedge-resections) was malignant melanoma. Genetic testing of the patient’s pulmonary metastases identified a p.V600E BRAF mutation, making the patient eligible for a targeted therapy with an oral BRAF inhibitor. In July 2012, the disease had progressed to stage IV with bone, pulmonary, liver and brain metastases, as revealed by lower back pain. The patient was then treated with the BRAF inhibitor vemurafenib (960 mg *per os*, twice daily). After four weeks of treatment, the patient’s general health status had improved and the lower back pain was less intense. Computed tomography imaging revealed stable disease, according to RECIST criteria (with a 25% decrease in the size of the metastases, Figure 1). Eight weeks after the initiation of vemurafenib treatment, the patient presented a deterioration in general health status and an elevation of liver transaminases and lactate dehydrogenase levels. The vemurafenib treatment was maintained but the dose was reduced to 720 mg *per os* twice daily. In view of the disease progression and the emergence of side effects and diffuse bone pain, vemurafenib was withdrawn after 12 weeks of treatment. Palliative care was administered. A month later, the patient died from the progression of metastatic disease.

### Molecular analysis

**Figure 2:**
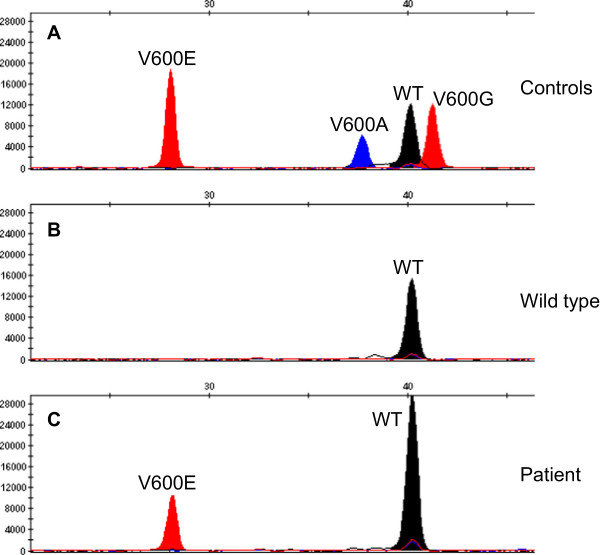
**Primer extension and fragment analysis.** Representative electropherograms of BRAF primer extension products, showing mutated and wild type controls **(A)**, tumor DNA from a melanoma patient without a *BRAF* mutation **(B)** and the patient’s tumor DNA with a p.V600E mutation **(C)**.

The initial diagnostic testing for *BRAF* mutations was performed by primer extension reaction (*BRAF* mutation analysis reagents, Applied Biosystems, CA, USA). The extension primer specifically targets the second nucleotide in codon 600 of *BRAF* gene, enabling the detection of p.V600E (G**T**G > G**A**G), p.V600G (G**T**G > G**G**G) or p.V600A (G**T**G > G**C**G) mutations but not mutations that also involve nucleotides at positions 1 or 3 (such as p.V600K (**GT**G > **AA**G) and p.V600D (G**TG** > G**AT**)). The primer extension products were separated by capillary electrophoresis on a 3500 xL Dx Genetic Analyzer (Applied Biosystems, CA, USA) and then underwent fragment analysis using Gene Mapper v4.1 software (Applied Biosystems, CA, USA). As shown in Figure 2, a peak corresponding to the p.V600E mutation (a thymine to adenine substitution at position 1799 (c.1799 T > A)) was detected in the patient’s tumor cells.

The presence of the p.V600E mutation was confirmed by bidirectional sequencing of *BRAF* exon 15 using an ABI PRISM Big Dye Termination Cycle Sequencing kit v1.1 (Applied Biosystems, CA, USA), a 3500 xL Dx Genetic Analyzer and Sequencing Analysis software v5.4 (Applied Biosystems, CA, USA).

**Figure 3:**
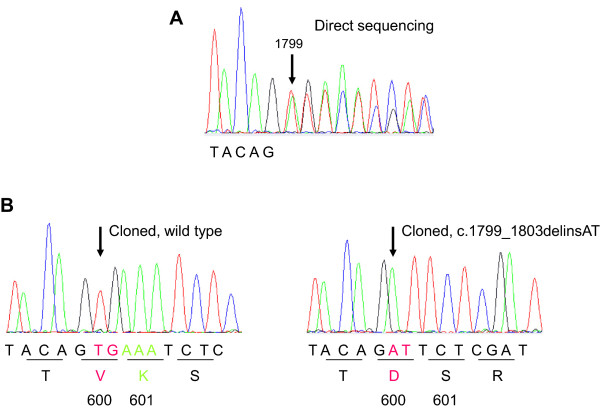
**Sequence analysis of** ***BRAF*** **exon 15.** Electropherograms from direct sequencing of the tumor’s genomic DNA **(A)**, displaying an overlapping pattern starting from nucleotide 1799 (indicated by an arrow) and cloned PCR products **(B)** showing the wild type allele (left) and the c.1799_1803delinsAT mutation (right) that leads to amino acid substitution and deletion (p.V600_K601delinsD).

**Figure 4:**
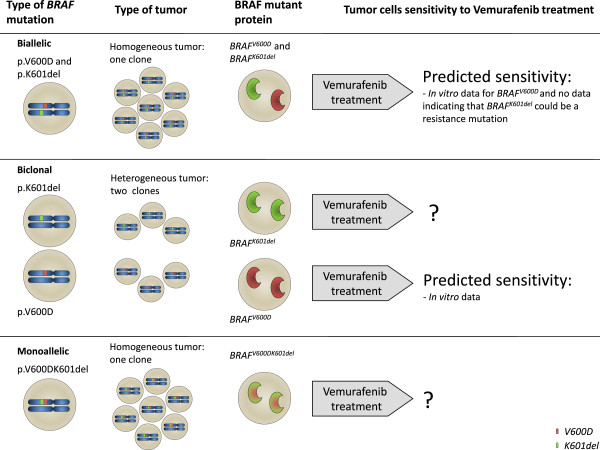
**Schematic representation of monoallelic, biallelic and biclonal mutations and their therapeutic consequences.** Mutations identified by molecular biology analysis of a DNA sample may reflect three distinct molecular events in tumor cells within a single tumor.

Surprisingly, sequence analysis revealed that the mutation was more complex than a single nucleotide substitution (Figure 3A). The electropherogram showed an overlapping pattern starting at position 1799, which most probably indicates a heterozygous deletion and/or insertion. By comparing the overlapping sequence with the *BRAF* gene’s reference sequence (NM_004333.4), we found that the mutation corresponds to c.1799_1803delinsAT (p.V600_K601delinsD) when expressing using Human Genome Variation Society-approved nomenclature [20]. At the protein level, this results in substitution of the valine (V) at position 600 by an aspartic acid (D) and the deletion of the lysine (K) at position 601 (*BRAF*^*V600DK601del*^). However, the identification of a dual mutation in genomic DNA extracted from a pool of multiple cells does not necessarily mean that the two mutations are carried by a unique allele; they may correspond to two alleles in a single tumor clone or even to two individual tumor clones. This distinction is important because it would probably result in a significant difference in the therapeutic response to vemurafenib (as summarized in Figure 4).

In order to characterize each of the alleles and establish whether the mutation was monoallelic (*BRAF*^*V600DK601del*^) or biallelic (*BRAF*^*V600D*^ and *BRAF*^*K601del*^), we applied a TA cloning-based sequencing method. The polymerase chain reaction (PCR) products from *BRAF* exon 15 amplification were cloned into a pCR™2.1 vector with the TA Cloning® Kit (Invitrogen, CA, USA) and the inserts were sequenced using the M13 forward and reverse primers.

Comparison of the electropherograms (Figure 3B and C) showed that one allele was wild type and that the other was mutated. In the mutant allele, thymine (T) and guanine (G) at positions 1799 and 1800 had been respectively replaced by adenine (A) and thymine (T). The substitutions had been followed by an in-frame deletion of three adenines (AAA). Hence, the mutation was a monoallelic event that resulted in a protein product with a valine to aspartic acid substitution at position 600 and an in-frame deletion of the lysine at position 601, yielding the *BRAF*^*V600DK601del*^ mutant protein.

### In silico crystal structure analysis

At the time of our analysis, the complex *BRAF*^*V600DK601del*^ mutation had not been reported in the literature. Consequently, no information on the potential vemurafenib response for this mutation was available.

**Figure 5:**
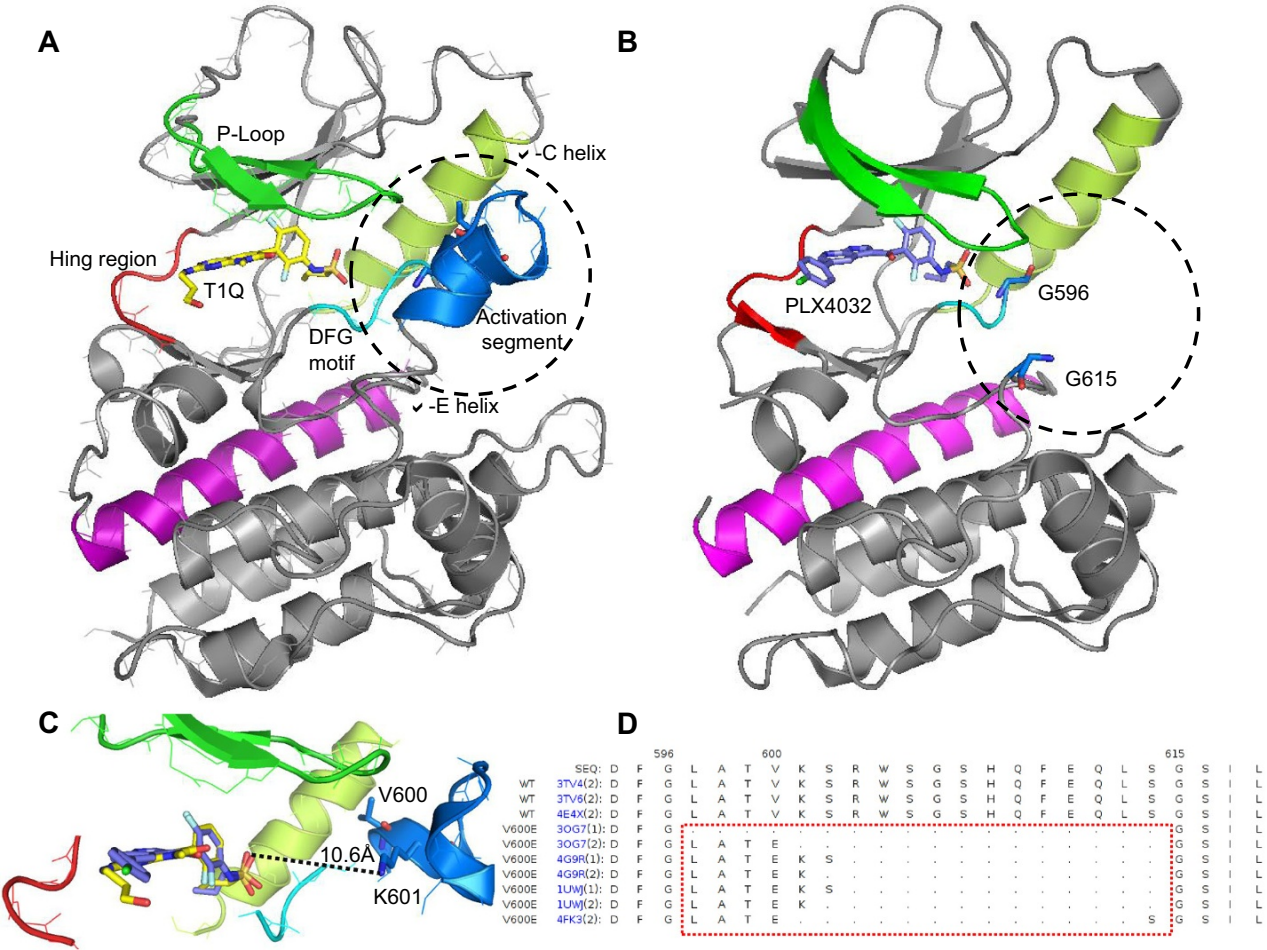
**Crystal structures of BRAF domain-inhibitor complexes. (A)** *BRAF* ^*WT*^ and T1Q (in yellow), **(B)** *BRAF* ^*V600E*^ and vemurafenib (in light blue), **(C)** comparison of binding modes of the two inhibitors, and **(D)** multiple sequence alignment of crystallized BRAF domains (amino acids residues lacking electron density are represented by dots. The BRAF domain structural regions are colored as follows: green: P-loop; red: hinge region; blue: activation segment; cyan: DGF motif; lime: α-C-helix; magenta: α-E-helix.

To establish whether p.V600_K601delinsD *BRAF* mutation-positive melanoma might be sensitive to treatment with vemurafenib, we performed a crystal structure analysis of BRAF proteins using Sybyl®-X software (Tripos, MO, USA). The following structures from the PDB database [21, 22] were used: 4E4X [23] and 3OG7 [14]. The Sybyl®-X software’s Biopolymer module was used to prepare, visualize, superimpose and compare the two crystal structures. Graphics were prepared with the PyMOL Molecular Graphics System (Schrödinger, OR, USA). As illustrated in Figure 5A and 5C, the activation segment (in blue) of *BRAF*^*WT*^ kinase domain holds the protein in an inactive conformation through association with the P-loop (in dark green) via predominantly hydrophobic interactions [13]. The V600 and K601 residues are located in the BRAF kinase domain’s activation segment. In the *BRAF*^*V600E*^ mutant, the medium-sized hydrophobic amino acid residue valine is replaced by the large, negatively charged glutamic acid residue; this disrupts the interaction between the P-loop and the activation segment (Figure 5B). In turn, this destabilizes the inactive conformation and promotes an active conformation with increased kinase activity [13]. In all *BRAF*^*V600E*^ kinase domain crystal structures, the atomic coordinates for activation segment region (K601-S614) have not been fully resolved by X-ray diffraction analysis (Figure 5D) - probably due to increased flexibility or even structural disorder caused by the mutation. The same can be expected of *BRAF*^*V600DK601del*^ mutant protein because of the physical-chemical similarity between aspartic acid and glutamic acid residues. This observation is also consistent with *in vitro* studies showing that (i) both V600E and V600D mutants have similar, elevated levels of kinase activity [13] and (ii) vemurafenib also potently inhibits the proliferation of melanoma cell lines expressing *BRAF*^*V600D*^ [24].

We next considered the impact of K601 deletion. When comparing the structure of *BRAF*^*V600E*^ cocrystallized in the presence of vemurafenib (PDB: 3OG7) with *BRAF*^*WT*^ cocrystallized with another selective inhibitor (T1Q) (PDB: 4E4X), it can be seen that the distance between K601 and the sulfonyl group of both inhibitors is more than 10 Å (Figure 5C) - suggesting that K601 does not participate in ligand binding. Moreover, the lack of an electron density map for the region including E600 and K601 in the vemurafenib-*BRAF*^*V600E*^ complex also suggests that neither of these residues is crucial for protein-ligand interaction. In view of the above arguments, we expected the *BRAF*^*V600DK601del*^ mutant protein to be sensitive to inhibition by vemurafenib.

## Discussion

Here, we reported on use of the BRAF tyrosine kinase inhibitor vemurafenib to treat a patient with a rare, complex *BRAF* mutation (p.V600_K601delinsD). To the best of our knowledge, only two other patients harboring the same mutation have been reported to date [7, 25]. Only one of the two patients was treated with vemurafenib, and the response was good [25]. The present case is therefore the second to display a positive response.

At the time of molecular diagnosis, the p.V600D mutation and the p.K601 deletion had been individually and independently described by different researchers [1, 4, 11, 26]; this suggested that the mutations were present on two different alleles, with the expression of two mutant proteins (BRAF^*V600D*^ and BRAF^*K601del*^). Furthermore, the presence of several distinct *BRAF* gene mutations in the same melanoma tissue sample has been reported [9, 27–29]. However, none of the latter reports distinguished between monoallelic and biallelic mutations or commented on their possible biclonal nature.

To establish whether our patient’s mutations were monoallelic (*BRAF*^*V600DK601del*^) or biallelic (*BRAF*^*V600D*^ and *BRAF*^*K601del*^), we performed cloning-based sequencing experiments. Molecular assessment of the heterozygosity of the mutations revealed that the two changes were monoallelic (Figure 3), resulting in a p.V600_K601delinsD mutant protein*.* Busser et al. and Heinzerling et al. did not perform allele discrimination experiments and so could not reasonably assume that the mutation resulted in *BRAF*^*V600_K601delinsD*^ [7, 25]. To the best of our knowledge, the present report is the first to show that both mutations occur within the same allele.

There are almost no literature data on the response to vemurafenib for tumors with complex *BRAF* mutations (probably because of their low incidence). In order to analyze the potential effects of the mutation on vemurafenib binding, we performed an *in silico* crystal structure analysis (Figure 5). Based on the structures of *BRAF*^*V600E*^ cocrystallized in the presence of vemurafenib and *BRAF*^*WT*^ cocrystallized with T1Q, we forecast that the protein encoded by the mutant allele *BRAF*^*V600_K601delinsD*^ would be sensitive to vemurafenib inhibition. The resolved crystal structures do not provide information on the impact of the K601del mutation on the flexibility of the activation segment. A more precise answer would require molecular dynamic simulations based on the model structure of the kinase domain bearing the complex mutation. This type of modelling might reveal the impact of the p.V600D_K601del mutation on activation segment flexibility and might enable estimation of the free energy of vemurafenib binding in the presence and absence of the K601 residue.

Nevertheless, our current modelling predictions are in agreement with the patient’s positive treatment response; vemurafenib therapy was associated with a rapid, objective, clinical response and 2 months of PFS. Our present observations are consistent with data shown in phase I, II and III clinical trials in which patients with an objective response to vemurafenib display disease progression two months after starting the treatment [15, 16, 30]. Our data suggest that the rare mutation *BRAF*^*V600_K601delinsD*^ is likely to induce a constitutively activated BRAF protein and should respond to BRAF inhibitors in the same way as the classic p.V600E mutation. In a more general way, performing predictive *in silico* structural analysis before the treatment is started could be helpful to evaluate the activating character of a mutation when the clinical efficacy is not well established. Moreover, this approach is currently being used in the French clinical trial AcSé crizotinib [31].

Interestingly, the p.V600_K601delinsD mutation was originally misidentified by the primer extension method as p.V600E. This is explained by the fact that the forward extension primer for V600E specifically targets the second nucleotide (T) in codon 600 (G**T**G), which in our case was also substituted for an A (G**AT**), as in the V600E mutation (G**A**G). However, in a retrospective study of 47 metastatic melanoma from our centre, we have compared the V600 mutation detection by molecular techniques with the *BRAF*^*V600E*^ mutant protein expression by immunohistochemical analysis using highly specific anti-*BRAF*^*V600E*^ monoclonal antibodies and no other cases of misidentified p.V600E mutation was found [32].

Since treatment of *BRAF*-mutated patients has a profound impact on disease and overall survival, the accurate diagnosis of rare *BRAF* mutations is crucial and must be correlated with documented reports on clinical benefits. However, this type of mutation might not be detected with certain mutation-specific assays [7, 33]; consequently, affected patients might be excluded from clinical trials with BRAF inhibitors or regular treatment with vemurafenib [16]. The choice between mutation-specific detection methods (which are rapid and highly sensitive, with detection of fewer than 5% of tumor cells in a normal background) and sequencing-based methods (which are extensive but also labor-intensive and/or less sensitive) is a recurrent dilemma for molecular biologists in the field of oncology. Indeed, care should be taken when choosing an approach for molecular testing in clinical practice; the technologies currently available for detecting the V600E mutation differ remarkably in terms of sensitivity and specificity - especially for rare mutations.

## Conclusions

Our experience suggests that (i) patients with a p.V600_K601delinsD mutation can be treated successfully with the oral BRAF inhibitor vemurafenib and (ii) molecular screening should include this type of mutation. New technological advances in massively parallel sequencing methods or digital PCR are currently being implemented in routine diagnostic laboratories and are being validated for safe use in clinical molecular oncology. These technologies will doubtless detect many more rare and/or complex mutations of unknown prognostic, diagnostic and/or therapeutic significance – thus highlighting the need for regularly and actively updated clinicobiological databases that provide the necessary justification for the therapeutic management of these patients.

### Consent

Written informed consent was obtained from the patient for conducting molecular analysis and this study was approved by the local ethics committee (Comité de Protection des Personnes (CPP) Nord-Ouest II, Amiens, France). Written informed consent was obtained from the relative of the patient for publication of this case report and any accompanying images. Copies of the signed consent forms are available for review by the Editor of this journal.

## Abbreviations

BRAF: v-raf murine sarcoma viral oncogene homolog B
COSMIC: Catalogue of somatic mutations in cancer
PCR: Polymerase chain reaction
PDB: Protein data bank
RECIST: Response evaluation criteria in solid tumors.
